# “You can't shoot another bullet until you've reloaded the gun”: Coaches' perceptions, practices and experiences of deloading in strength and physique sports

**DOI:** 10.3389/fspor.2022.1073223

**Published:** 2022-12-21

**Authors:** Lee Bell, David Nolan, Velu Immonen, Eric Helms, Jake Dallamore, Milo Wolf, Patroklos Androulakis Korakakis

**Affiliations:** ^1^ Department of Sport and Physical Activity, Sheffield Hallam University, Sheffield, United Kingdom; ^2^ School of Health & Human Performance, Dublin City University, Dublin, Ireland; ^3^ Department of Sport and Health Sciences, Technological University of the Shannon, Athlone, Westmeath, Ireland; ^4^ Department of Sports and Exercise, Haaga-Helia University of Applied Sciences, Vierumäki, Finland, United Kingdom; ^5^ Sport Performance Research Institute New Zealand (SPRINZ), Auckland University of Technology, Auckland, New Zealand; ^6^ Centre for Health, Exercise and Sport Science, Solent University, Southampton, United Kingdom

**Keywords:** training cessation, detraining effect, periodisation, reduced training, tapering

## Abstract

Deloading refers to a purposeful reduction in training demand with the intention of enhancing preparedness for successive training cycles. Whilst deloading is a common training practice in strength and physique sports, little is known about how the necessary reduction in training demand should be accomplished. Therefore, the purpose of this research was to determine current deloading practices in competitive strength and physique sports. Eighteen strength and physique coaches from a range of sports (weightlifting, powerlifting, and bodybuilding) participated in semi-structured interviews to discuss their experiences of deloading. The mean duration of coaching experience at ≥ national standard was 10.9 (SD = 3.9) years. Qualitative content analysis identified Three categories: *definitions, rationale,* and *application.* Participants conceptualised deloading as a periodic, intentional cycle of reduced training demand designed to facilitate fatigue management, improve recovery, and assist in overall training progression and readiness. There was no single method of deloading; instead, a reduction in training volume (achieved through a reduction in repetitions per set and number of sets per training session) and intensity of effort (increased proximity to failure and/or reduction in relative load) were the most adapted training variables, along with alterations in exercise selection and configuration. Deloading was typically prescribed for a duration of 5 to 7 days and programmed every 4 to 6 weeks, although periodicity was highly variable. Additional findings highlight the underrepresentation of deloading in the published literature, including a lack of a clear operational definition.

## Introduction

Athletes involved in competitive strength sports perform regular resistance training to enhance their athletic performance (1). The development of muscular strength is a key performance characteristic in strength-based sports such as powerlifting and strongman, where the ability to produce maximal force is a primary goal (2). Strength development also enhances mass-specific force generation, rate of force development, and impulse, and is, therefore, an important determinant of performance in maximal effort sports such as weightlifting, throwing, jumping, and sprinting (3, 4). However, whilst an improvement in muscular strength appears to benefit athletic performance, current evidence does not allow for definitive statements to be made in regard to the causal effect of strength on sports performance (5). In physique sports such as competitive bodybuilding, the goal for the athlete is to achieve both leanness and hypermuscularity (6), and competitors are judged on muscular appearance and proportionality rather than athletic performance (7). Therefore, the development of muscle hypertrophy is a primary focus of training (6–9).

To achieve a meaningful level of physiological adaptation that underpins performance, athletes typically participate in strategically-planned resistance training, organised in a cyclical manner relative to the competition schedule (10, 11). These cycles (often referred to as “mesocycles”) often involve periods of intensive training designed to stimulate an adaptive response (12, 13). However, since an increase in fatigue is a consequence of continuous, progressive training, periods of reduced training demand are also planned and might be necessary to facilitate physiological adaptations by reducing fatigue and mitigating the risk of maladaptation (14, 15). Without sufficient restoration, longer-term decrements in performance indicative of non-functional overreaching can occur due to overtraining (16). Adequate rest from strenuous exercise is considered the most effective intervention to reduce the risk of overtraining and to accelerate recovery (17). As such, a successful strength or hypertrophy training programme should emphasise the appropriate adaptation whilst being cognisant of the effects of prolonged or excessive training demand. Consequently, traditional periodised training incorporates periods of reduced training demand designed to reduce fatigue, avoid the deleterious effects of prolonged high training demand, and facilitate meaningful physiological adaptation (12, 14, 15). Such periods of reduced training can take place within the overall training macrocycle (e.g., during the off-season), during the training mesocycle (e.g., a lower training demand week), or within a training microcycle (e.g., lower demand training sessions or days off) (11).

Phases of reduced training can occur immediately prior to competition (i.e., tapering) or at select periods during the overall training programme. Phases of reduced training not performed immediately prior to competition have been referred to as restitution/recovery microcycles (14), recovery weeks (18), unloading weeks (19, 20), regeneration microcycles (21), and deloading (22). In general, these phases aim to facilitate physiological adaptation, reduce the risk of overtraining (20) and assist in reducing training monotony that can occur during the competitive season (23). As a form of reduced training, deloading refers to the purposeful reduction in overall training demand with the intention of enhancing preparedness (18, 22). Deloading also aims to mitigate the risk of physiological maladaptation and injury (19) and is considered an important “fatigue management tactic” that enhances the potential success of the overall programme (14). Deloading occurs sporadically throughout the overall training programme (24, 25) and is likely to occur following periods of prolonged or challenging training, such as planned overreaching, or at the end of a training mesocycle (16, 25–27). The most frequently reported duration for a deload is one week (i.e., microcycle) but ranges from a singular training session to two weeks (14, 28). Whilst the general concept of deloading seems to be well established, little is known about how the necessary reduction in training demand should be accomplished. According to the (albeit disparate) available literature, a reduction in training demand could be achieved by altering the number of weekly training sessions (29), movements/muscle groups trained (25, 30), the number of weekly working sets per muscle group (18, 22), repetitions performed within a set (31), percentage of one-repetition maximum (1-RM) (29, 32), or proximity to muscular failure (30). However, it is currently unclear how these variables should be organised and manipulated for adequate recovery without inducing a loss of physiological adaptation and detraining effect.

Whilst deloading is likely to occur at the end of each training mesocycle (18, 24, 29), tapering occurs specifically in the days/weeks prior to competition (2) and is common practice in strength sports (26, 33–37). The aim of the taper is to facilitate “peaking”, where the athlete achieves optimal physiological performance prior to competition due to a reduction in fatigue and an increase in preparedness (35, 38). Previous literature highlighted that 87%–99% of competitive strength athletes incorporate a taper into their programme prior to competition (34, 36, 37). Conversely, competitive physique athletes (e.g., bodybuilders) do not incorporate tapers into their resistance training programmes. Instead, physique athletes typically maintain a similar resistance training stimulus in the final “peak week” prior to competition with only minor adjustments, whilst simultaneously increasing (sometimes decreasing or completely eliminating) fat loss-focused cardiovascular training sessions (7, 30, 39). In the final days prior to contest day, physique athletes might also alter macronutrient and energy intake, hydration status, and muscle glycogen levels to enhance muscularity and achieve peak aesthetic condition (40). Slight alterations to training during peak week can include staying further from muscular failure, completing training days earlier in the week, and avoiding exercises which train muscles at long lengths to reduce muscle damage; however, these modifications are not to reduce fatigue, but because muscle damage can interfere with muscle glycogen synthesis (40). The omission of a resistance exercise taper in physique sports is likely due to the emphasis on peak aesthetic condition rather than physical performance (30, 41, 42).

Based on the current evidence, deloading and tapering can be differentiated primarily by their position within the overall training programme, as well as their overall objective. However, the way in which training is adjusted during both phases shares several similarities (2, 18, 24, 29), which might lead to misinterpretation. Indeed, previous commentary (38) has used the terms deload and taper interchangeably, stating that the taper does not only take place prior to competition, but might also occur during maintenance phases of training or intermittently during the training programme. There is, therefore, not only a clear need to develop training guidelines to optimise deloading in strength and physique sports, but also to explore how deloading might differ conceptually from tapering.

Whilst there is a paucity of research within the deloading domain, there are several published YouTube videos providing educational content on deloading practices (some of which have gained 120–560 thousand views at the time of writing), as well as 86.3 thousand #deload hashtags on Instagram, suggesting that deloading as a training tool is garnering attention online and in practical training environments. This research project developed out of a series of discussions between members of the research team, all of whom are strength/physique practitioners, sport and exercise science researchers or academics with an interest in strength and conditioning. The research question originated from a shared concern that there is a dearth of empirical research on the topic, even though deloading is a common training practice in strength training environments. By exploring the perceptions and lived experiences of coaches who utilise deloading in their practice, it is hoped that the overall understanding of the topic will be improved. As such, information disseminated in this research will inform future empirical research in this area, where the development of real-world deloading protocols and training practices will no doubt be of key importance. Previous research using semi-structured interviews has investigated tapering practices in strength sports (43, 44). However, to our knowledge, this is the first research study that explores deloading practices performed within strength and physique sports from the perspective of the coach. The purpose of this research is, therefore, to determine current deloading practices in competitive strength and physique sports to (1) provide a framework of existing practice for strength and physique practitioners who intend on implementing deloading within their training programmes, (2) assist clinicians in the development of “real world” protocols for use in future experimental research, and (3) to identify the similarities and differences between deloading from tapering.

## Methodology

### Approach to the problem

A qualitative descriptive research design was adopted as it allows a straightforward presentation of the information collected, organised in a way that best fits the data and what is most relevant to the anticipated readers (45). Whilst descriptive, this design allows for a critical examination of the deloading phenomenon and is suitable for research questions focused on discovering the who, what, and where of phenomena, particularly in areas where little is known about the topic under investigation (46). Interview data were analysed using directed qualitative content analysis described by (47). Qualitative content analysis is a research method for making replicable and valid inferences from data to the context of their use, with the purpose of providing new insights, understanding particular phenomena, and informing practical guidelines (48), and was therefore considered appropriate for this research.

### Participants

After institutional ethical approval (ER38311849), 18 male participants were recruited using a convenience sampling approach that recruited eligible participants on a first-come, first-served basis. Participants represented a cross-section of strength and physique sports: weightlifting (*n* = 3), powerlifting (*n* = 12) and bodybuilding (*n *= 10). Some participants (*n *= 7) represented more than one sport (see Table 1 for a detailed descriptive profile of each participant). The sample size was convenience-based and justified based on feasibility expectations given the researchers' access to the sample population, i.e., a resource constraints-based justification (49). The mean duration of coaching experience at ≥ national standard was 10.9 (SD = 3.9) years. Fifteen participants had additional experience competing as an athlete at a minimum of national level in their respective sport. Education level ranged from no academic degree (*n =* 2) to Doctor of Philosophy (*n = *10). Participants possessed a range of relevant sport-specific governing body certifications, with some holding additional Personal Fitness Training or Strength and Conditioning accreditations (e.g., National Strength and Conditioning Association). Each potential participant was screened for eligibility, and informed consent was obtained prior to taking part in the interview according to the principles of the Declaration of Helsinki (50).

**Table 1:** Descriptive characteristics of participants.

Participant	Sport(s)	Experience category	Experience (years)	Experience (coaching level)	Country
1	Bodybuilding, Powerlifting	Coach	10	International	USA
2	Powerlifting	Coach	10	International	CA
3	Powerlifting, Weightlifting	Coach	15	International	USA
4	Powerlifting	Coach, Athlete	6	International	NZ
5	Bodybuilding, Powerlifting	Coach, Athlete	13	National	USA
6	Bodybuilding, Powerlifting	Coach, Athlete	17	International	USA
7	Bodybuilding	Coach, Athlete	10	International	AUS
8	Bodybuilding	Coach, Athlete	9	International	USA
9	Bodybuilding	Coach, Athlete	13	International	USA
10	Bodybuilding	Coach, Athlete	20	International	USA
11	Bodybuilding	Coach, Athlete	14	National	USA
12	Bodybuilding	Coach, Athlete	7	International	UK
13	Bodybuilding, Powerlifting	Coach, Athlete	8	International	USA
14	Powerlifting	Coach, Athlete	10	International	USA
15	Powerlifting	Coach, Athlete	10	International	USA
16	Powerlifting	Coach, Athlete	6	International	USA
17	Powerlifting, Weightlifting	Coach, Athlete	6	International	NZ
18	Powerlifting, Weightlifting	Coach, Athlete	12	International	USA

AUS, Australia; CA, Canada; NZ, New Zealand; UK, United Kingdom; USA, United States of America.

### Data collection

A semi-structured interview approach was selected as the data collection method as it provides an opportunity for comprehensive but flexible information collection, where opinions can be complex and nuanced (51–53). Semi-structured interviews have previously been employed within strength and conditioning research where deep exploration of perceptions and attitudes towards training practice is the topic of interest (16, 44, 54–56).

Before data collection, an initial semi-structured interview guide was created by three researchers (LB, PAK, DN) and shared with the whole research team for feedback. Each researcher involved in the development of the guide has previous experience in qualitative research using semi-structured interviewing. The interview guide was refined through pilot interviewing of three participants that met the inclusion criteria. Piloting provided an opportunity to review the initial interview guide and resulted in further refinement of interview questions (57). None of the participants used for piloting were included in the final sample. The final version of the interview guide reflected the aims of the research and facilitated the collection of rich data that remained focused on the study objectives, but also permitted additional questioning through relevant dialogue between participant and interviewer (51). All interviews were conducted by one researcher (PAK). The full interview guide is located in Supplementary Appendix S1.

Participants were invited to a single online interview (mean duration 0:34:16; SD = 0:12:58 min) using European Union General Data Protection Regulation-compliant communication software (Google Meet). All interviews were conducted between November 2021 and February 2022. The recorded audio from each interview was exported to a password-protected hard drive using video converter software (Wondershare Technology Co, Shenzhen, China). At this stage, all data files were anonymised, with a unique identification number assigned to each participant chronologically based on the order of the interview to protect anonymity (see Table 1). Audio files were then exported to an online artificial intelligence transcription service (Otter.ai, Los Altos, California), where they were checked for accuracy by two researchers (JD, PAK). During each interview, participants were encouraged to answer questions comprehensively and to provide accurate and practical experiences where possible. The interviewer sought to conduct interviews in a relaxed manner, and questioning was approached flexibly (58). Follow-up questions were used to collect open-ended data, explore relevant additional lines of enquiry, and delve deeply into participants' feelings and beliefs about the research topic (57).

Direct, anonymised quotes (using the participant's unique identifying number assigned in Table 1) were used within the results section of this research study to illustrate discussion points and contextualise each category of information. Additional words are placed in parentheses, where required, to reduce ambiguity, clarify the intended meaning or provide further context. Punctuation has been added to quotations to reduce ambiguity where relevant.

### Data analysis

The main steps of the qualitative content analysis were as follows: (1) preparation, which involved examination of the interview data; (2) organising, which involved coding and grouping the data into conceptual categories; and (3) reporting a summary of the findings and their implications for practice, education, and future research (47). Three researchers (PAK, LB, DN) were involved in the process of data analysis. In the initial stage of analysis, each researcher was randomly allocated *n *= 6 interview transcripts and audio recordings, where the manifest content was analysed independently. Transcripts were read, re-read, and initial “points of interest” relevant to the research question were highlighted. Next, an unconstrained categorisation matrix (47) was created, where initial categories were created within its bounds using content-characteristic words. Data were then grouped into category headings, which were updated and refined through each stage of analysis. Lastly, subcategories were created to manage the large volume of data within each category and to assist with publicising of results. Throughout the data analysis process, a Google Docs file was used by the researchers as a means to collaborate, share ideas, refine codes and themes, and to provide a transparent audit trail of decision making. As a deductive approach based on an earlier theory was used, the results were presented from general to specific (59, 60).

### Methodological rigour

High-quality qualitative research is contingent upon trustworthiness and transparency (61). To achieve the desired level of quality in qualitative research, the researcher must acknowledge how the relationship between the interviewer and participant might influence the construction of knowledge (62). *Reflexivity* describes the intersecting contextual relationships between the participant and researcher, and this serves as a tool to achieve trustworthiness and transparency (63). During each phase of data analysis, peer debriefs were held to discuss data, corroborate ideas, and verify decision-making (61). A detailed audit trail was maintained to track changes or modifications at each stage of analysis and to establish methodological rigour (64). It should be noted that the research team applied their interpretation to the data based on their knowledge and experience (e.g., resistance training programming, periodisation). We have attempted to demonstrate the conceptual and theoretical decision-making process that underpinned our qualitative research methodology by detailing the steps taken at each stage of the data collection and analysis process.

## Results

A central concept of deloading was organised into three main categories: *definitions, rationale,* and *application*. Additional subcategories were developed to provide structure to main categories and to further organise the data into meaningful patterns whilst demonstrating a hierarchy of meaning. Table 2 provides a schematic representation of categories and subcategories.

**Table 2:** Summary of categories and subcategories.

Category	Subcategory
Definitions	Training demand
	Differentiating the deload from the taper
	Interchangeability
Rationale	Fatigue management and recovery
	Progression
Application	Training volume
	Intensity of effort
	Training Frequency
	Duration
	Exercise variation
	Individualisation
	Proactive versus reactive
	Periodicity

### Definitions

In this category, participants defined deloading and elucidated how it could be distinguished from the taper. Three subcategories were developed to help contextualise the information provided by participants in this category: *training demand*, *differentiation*, and *interchangeability*. Overall, participants defined deloading as a point within the overall training programme where training demand was intentionally and systematically reduced. Tapering and deloading were distinguished solely based on their position within the overall training programme: the taper takes place prior to competition, but the deload could occur at any point within the training programme. However, some participants used the term taper when referring to microcycles of reduced training demand that occurred earlier in the training calendar, demonstrating the interchangeable use of the terms taper and deload.

### Training demand

The first subcategory addressed the intentional manipulation of training. Overall, participants described the deload as an “*easy training”* strategy*,* a *“reduction in the difficulty in training”*, and where training emphasised “*cutting back on the total amount of workload being done”*.

“An intentional period of reduced training difficulty” (4).

“A period of intentionally reduced training stimulus” (10).

“A temporary, intentional reduction in workload” (5).

### Differentiating the deload from the taper

In this second subcategory, participants described how they distinguished the deload from the taper. For most, the difference was based on positionality relative to the competition; whereas the taper occurred directly prior to the competition, and the deload occurred intermittently across the overall programme.

“A taper would specifically be prior to a competition” (13).

“A deload is something you do as a part of the training process. A taper is something you do immediately prior to competition” (3).

“I guess [they're] a similar thing, just potentially with a different outcome” (17).

For participant 23, what distinguished the taper from deloading was not based on positionality within the training programme itself, or in the manipulation of training variables, but in planning.

“A taper is planned, whereas a deload is not necessarily planned” (15).

### Interchangeability

In the final subcategory, interchangeability between the terms tapering and deloading was revealed. Whilst several participants clearly differentiated the taper from the deload by positionality or objective, others appeared to use the term *taper* and *deload* synonymously.

“Tapering versus [the term] deloading is often used interchangeably” (15).

At times, participants used the term *taper* when referring to microcycles of reduced training demand that did not occur prior to competition or peaking phases of training.

“We probably need to run some kind of taper, just to give you a chance to catch up recovery-wise” (6).

“So just say, here's our build-up and volume, we're gonna taper after and then we're gonna get going on the actual developmental work” (2).

For participant 3, duration was the determining factor when referring to reduced training demand as a taper or a deload.

“So, when I talk about deloading, it's typically a day or a week at most, if we're talking though, like a two to three to a four-week reduction in volume, I'm probably gonna call that a taper” (3).

### Rationale

In this category, participants described the underpinning objective and rationale behind deloading. Two subcategories were developed to manage the information provided by participants: *fatigue management and recovery* and *progression.* Overall, fatigue management was a key objective for the implementation of deloading. In this sense, the deload was viewed as a preventative, prophylactic aspect of training with the goal of dissipating physiological and psychological fatigue. Moreover, participants considered the deload to enhance progress and preparedness for the next phase of training by facilitating physiological adaptation whilst mitigating the risk of injury and illness. Additionally, participants considered recovery and regeneration to be an important objective of the deload, achieved through the reduction in fatigue caused by reduced training demand.

### Fatigue management and recovery

In this subcategory, participants indicated that the primary objective underpinning deloading was the management of fatigue. The deload was considered the point in training where “*some of the strain”* of training could be reduced, the focus of training prioritised “*physical and mental regeneration”,* and an opportunity for the athlete to “*wash out”*, “*switch things up”,* “*reset”* or “*have a little break”*.

“Deloads are a strategy for fatigue management” (4).

“A period of time where we are looking to achieve a reduction in fatigue” (12).

“The aim is to decrease either true physical fatigue marked by a decrease in performance prior to that period, perceived physical and/or mental fatigue. So, I would say, just a period of time that allows the athlete to reset to a baseline in which they feel ready again to push training to progressively overload” (18).

Due to the importance of fatigue management, the deload was considered to be an important facilitator of recovery. In this sense, the deload was organised in a way that promoted physiological and psychological well-being.

“A strategic period of low training intensity and low volume with a specific function of facilitating recovery” (7).

“It's an opportunity to recover” (12).

“A specific function of facilitating recovery” (7).

### Progression

The second objective of deloading highlighted by participants was to achieve progression. In this sense, progression referred to the improvement of physiological adaptation that had a meaningful effect on competition performance.

“Periodic reductions in training, volume and intensity are necessary for making progress” (10).

Deloading was linked to progress in three distinct ways: (1) that it facilitated physiological adaptation and therefore enhanced select markers of athletic performance, (2) that it reduced the risk of maladaptation such as illness or injury, and (3) that it reduced staleness and potential for burnout.

“We're able to control the rate of progress and make sure that we are getting stronger efficiently, but also staying healthy while doing so” (16).

“(The deload) allows the athlete to reset to a baseline in which they feel ready again to push training to progressively overload” (18).

“A huge reason (to deload) is to continue progress while reducing the risk of injury… from a psychological standpoint, preventing burnout and allowing them to continue to enjoy the process” (8).

However, not all participants agreed that deloading was necessary to ensure consistent training progression.

“Deloads are not necessary for making progress, there are very few things that I would say are absolutely necessary to make progress. Deloads are not one of them” (4).

### Application

In this category, participants described the manipulation and organisation of training during the deload. Due to its multidimensional nature, several subcategories were developed to contextualise the information located within this category: *training volume, intensity of effort, training frequency, duration, exercise variation,* and *individualisation*. A reduction in training volume was viewed as the most important modification to programming during the deload. This reduction was achieved *via* decreased repetitions per set, number of sets per training session, number of training sessions per week, or through a targeted approach where deloading of specific muscle groups or exercises occurred.

### Training volume

Participants conceptualised *training volume* as a reduction in either the number of repetitions completed per set or the number of sets per training session/week. For many participants, a reduction in training volume was an important aspect of the deload, as prolonged high volume was considered “*the largest contributor of fatigue”*. Participants emphasised the importance of individualisation when modifying training volume. Consequently, participants described the “*considerable”* and “*significant”* reduction in training volume in a broad sense, which ranged from 25% to > 50%. It appeared that those involved in physique sports were more conservative in alterations in training volume compared to strength coaches. Strength coaches were more likely to preserve training volume in the competition lifts and reduce “*accessory*” training volume, whereas physique coaches had a more flexible, general approach to training volume reduction, achieved through a decrease in sets for most or all exercises.

“With my bodybuilding athletes, we reduce their volume, roughly [by] 25%” (13).

“We maybe back off volume, pull volume back by two-thirds, half, something like that” (11).

“I would reduce total volume by something like 30 to 40%” (4).

“An example of a deload might be somebody cutting their volume back by 50% or dropping their intensity back severely” (16).

“A deload is reducing volume by more than 50%” (2).

“Generally, the volume of training is brought down by about half if not more” (10).

“We generally reduce their training volume with a specific focus on reducing accessory volume” (5).

“Number of sets is generally the first thing [that I reduce]. Like, if it's a standard hypertrophy type programme, number one is always going to be [to] reduce sets by at least 25%, a reduction that's across the whole training week” (1).

A reduction in training volume during the deload was not always conceptualised as a global training modification. Instead, the reduction in training volume could be achieved through a reduction in specific muscle group exercises or, in the case of strength sports, a reduction in specific exercises. In this sense, participants elucidated a *targeted* approach to deloading.

“Sometimes a deload is done specifically to a muscle group because the muscle group has been mildly injured and needs several days of recovery” (10).

“Deloads for me are kind of movement specific. So, like, we can deload squat and deadlift, but you know, allow bench press to kind of continue on as normal. Or even if (the) deadlift is going well I can deload the squat pattern” (14).

### Intensity of effort

Here, participants described how alterations in *intensity of effort* might be applied during the deload. Overall, changes in intensity of effort were closely linked to changes in training volume, and participants often described deloading as the synonymous management of training volume or intensity of effort, or in many cases, both. Participants conceptualised alteration in external changes to repetition maximum as changes in “*load”, “intensity”, and “percent rep max”.* When discussing alterations in internal measures of perceived effort (*“going to failure”*), it was common for participants to refer to ratings of perceived exertion (RPE) and repetitions in reserve (RIR).

“It's taking a step back from both volume and intensity” (2).

“A deload is a period of training, where you reduce intensity or volume, or both” (1).

“A period of time with reduced training volume, and/or training intensity” (4).

“I'd say in most cases, a deload for me is both a reduction in training volume and a reduction in intensity” (14).

Participants achieved a reduction in the intensity of effort through either change in external load or through internal training demands such as alteration in proximity to failure. In some cases, deloading was achieved through a concomitant reduction in external and internal measures.

“Typically, what I would do is reduce intensity, both peak and average intensity, by about 10%” (4).

“I usually tell them that all training sets should be terminated with at least four repetitions in reserve” (5).

“For me, oftentimes in the sense of absolute loading rather than just RPE… but typically a combination of both” (17).

Several participants considered alterations in intensity of effort secondary to reductions in training volume. In this sense, a reduction in intensity of effort was only applied to the deload if reduced training volume had not resulted in the desired decrease in fatigue.

“If they're really pulled back (I reduce) load a little bit too, so they're not going to failure. If they're really beat up, maybe we really pull back load, or if they're really beat up, that's where I would even err on the side of taking just some more days off” (11).

“I would like to recommend an athlete maintain their intensity - load - through that time, generally speaking. But if I had someone who came in with some connective tissue issues… I would probably vote for a reduction in intensity as well” (7).

However, some participants considered a reduction in training intensity as a primary aspect of the deload. Some favoured a reduction in intensity of effort over training volume. The reduction in intensity of effort was viewed flexibly and could be achieved in many ways.

“[For the] deload, I often back off intensity” (3).

“The way I've always programmed it is [that] you do the same reps, but we're just taking off a certain percentage so that's how I would categorise the deload” (6).

“For me, it's predominantly (with a deload), the intensity that”s going to be my big thing. So, I'm going to generally scale that back considerably. Sometimes it will be by nature of exercise choice, rather than necessarily by RPE, or reps and reserve. So that's probably one thing as a caveat there is, it's going to be, for me, oftentimes in the sense of absolute loading rather than just RPE… but typically a combination of both” (17).

### Training frequency

Participants conceptualised training frequency as the number of training days undertaken during deloading. Overall, participants aimed to maintain training frequency during the deload but would consider reducing the number of training sessions if the athlete presented excessive fatigue.

“I very rarely mess with frequency in terms of a deload” (13).

“I'm not completely opposed to taking time off as part of a deload or even training fewer days that week, you know, a couple of light workouts to training fewer days, you know, something like that I'm totally fine with” (11).

### Duration

In this subcategory, participants described the duration of the deload. Overall, participants agreed that the precise duration would be “*individualised”* to the athlete, but for most participants, the “*typical”* deload would be one week.

“The typical duration of a deload is one week” (10).

“It's usually one week” (5).

However, some participants suggested that shorter deloading periods might be more suitable due to decreased risk of detraining effect caused by loss of physiological adaptations.

“The deload doesn't necessarily have to be a whole week, right? It can be a few days” (15).

“I generally don't like to do more than six days… and a deload in excess of that likely means we're going to be reversing some of the adaptation. I want the deload to be long enough to recover, but not long enough to reverse that adaptation. So, I like a six-day deload” (7).

### Exercise variation

Deloading was viewed as an “*opportunity”* to vary exercise selection by most participants. However, the rationale for such variation was different between coaches. For some, changes in exercise selection provided the opportunity to reduce training monotony by “*changing things up”*, particularly for athletes that “*enjoy a lot of novelty”.* For others, exercise variation served to reduce the potential for overuse injuries and encouraged recovery by “*removing spinal loading exercises”.* For all participants, though, the choice of exercise still had to achieve “*carryover”* and “*purpose”* relative to the goal of the overall training programme.

“[The deload is] absolutely a chance to add new movements, as long as the movements have a purpose, you know, from the athlete”s training… or something (just) what we want to try” (14).

“If somebody was doing a barbell back squat, you know, maybe that day, on that deload week, they're just doing Smith squats, or a machine hack or something like that” (8).

“I also think of it as a kind of a transition period or a potential washout period…in which you're introducing some novelty for the sake of novelty to almost desensitise or reset the training stimulus if you feel that, that the performance has plateaued, or that the training response has been blunted” (18).

Not all participants would vary exercise selection during the deload. For some participants, it was intensity of effort and training volume that were favoured above exercise selection. This was, in part, based on the competition level of the athlete, with high-performance strength athletes likely to maintain specific exercises within the deload but at a lower demand.

“If an athlete is just training for fun, I'll give them more novelty and variation, whereas if an athlete is training for a world championship, we're very focused” (2).

“It'd be volume, intensity, and effort [that I would adapt]… effort being, you know, rate of perceived exertion.. those are really the only three that I typically mess with” (13).

“I might want to give the body a bit of a break, but I might not want the strength to disappear too much. So that's when I might keep things a little bit more specific… and maybe they'll do a single or triple or something. But the RPE might only be around, say, a six or seven, rather than eight or nine” (28).

For participant 14, novel exercises might be counterproductive during the deload due to the increased risk of exercise-induced muscle soreness.

“Because of the repeated bout effect, we may end up with actually more soreness using a brand new movement, which kind of defeats the purpose of the deload anyways” (14).

### Individualisation

In this subcategory, participants described the role of individualisation when organising training during the deload. Individualisation was contextualised by participants in two ways: (1) the need for undertaking a deload is highly variable between athletes, and (2) the manipulation of training variables during the deload requires individualisation. For all participants, adjusting the deload to suit the needs of the individual athlete was more important than following a generic approach or “*rigid”* system.

“I try to be as individual as I can” (17).

“(There”s) a high degree of variability from person to person. I've seen some clients who, you know, when we go for that sixth week in a row without a deload, it seems like we're really pushing it. And other clients who, even after 12 weeks of hard training, just simply don't seem to really need one yet” (5).

The individualised approach to deloading was multifactorial, with several factors influencing how the deload was organised and prescribed. These factors included the level of ability, the personality of the athlete, the importance of competition, and chronological and training age.

“I try to be as individual as I can. So sometimes, obviously, knowing how old someone is, or their training age, or their competitive history, it obviously impacts things. But you might be looking at it more through the lens of that lifter more than necessarily their defining characteristics. The other things that define them as lifters, whether that be, you know, novice or really experienced, it's kind of like, what does that person tolerate? And what does that person enjoy? [This] is still one of the big factors, I kind of think about in all of these regards” (17).

“If we just have an athlete like an intermediate (level) athlete deep in the offseason, there's far more flexibility with deload” (14).

“I mostly change [the deload] based on the athlete feedback rather than on their level” (4).

Whilst participants revealed several factors that had an influence on deloading, biological sex was not considered to be a determining factor by any participant when individualising the deload.

“Biological sex is not something that influences the way I structure a deload” (5).

“I usually do the same [deload] for either gender. I mean, both genders can overreach and get to a point where they need to back off. As far as age goes, though, you know, some of my older clients are more experienced lifting clients, those are the ones where I would be more likely to say, hey, let's take five days off, let's take seven days off, just go train two days this week, or go train three days this week to take the rest of the week off” (11).

“I don't think I've made any specific changes based upon someone's gender directly, like, you know, in the aspect of a deload period” (17).

### Periodicity

Periodicity refers to how frequently participants would prescribe a deload during the overall training programme. In this subcategory, participants elucidated that periodicity of the deload would be highly individual, with coaches suggesting that athletes would undertake a deload “*every few weeks”*. The exact periodicity described by participants was broad (ranging from 3 to 12 weeks). Importantly, deloading would often take place at regular pre-determined time points within the training programme but also could be integrated reactively at any point where the athlete exhibited symptoms of excessive or prolonged fatigue that negatively impacted training performance.

“I would say if an athlete hasn't deloaded for three, five weeks, even if their training is feeling fine, I'm going to give them one anyways” (14).

“I'd say between four to six weeks, on average” (2).

“But I would say, probably anywhere from six to 12 weeks, you know, I would say somewhere in that realm” (7).

### Proactive vs. reactive

Participants described the implementation of deloading as either a proactive (pre-determined) or reactive, “*autoregulated”* aspect of training. In this sense, participants elucidated the advantages and disadvantages of deloading at pre-planned time points within the training programme versus “*taking the deload only when needed”*. A small number of participants favoured the use of pre-planned deloading:

“I typically pre-plan deloads for my athletes” (4).

“If I'm taking on a new athlete I pre-schedule them out, like the fifth week, just to check in and say, okay, we're still figuring each other out, let's go ahead and take a deload” (1).

Others avoided pre-planned deloading, favouring a more reactive, autoregulated approach:

“You're seven weeks into this plan, and you're still getting stronger. Why would we stop and deload? You're telling me you're feeling good, energy is good, like, we don't need to stop yet. We will at some point, you're not gonna be able to go forever, but let's keep going” (11).

“I don't think planned deloads are necessary” (18).

However, most stated that a “*flexible”* approach that combined reactive and proactive deloading would be optimal, with pre-planned deloads acting as “*checkpoints”* to assess the need for deloading rather than compulsory changes to programming. At times, participants described a range of factors that might influence the use of proactive and reactive deloads. These ranged from the competitive level and experience of the athlete and non-training commitments (work stress, holidays etc.) to previous knowledge of how the athlete best responds to training, e.g., “*you just know”*.

“Typically, when my athlete deloads [it's] because they've got some sort of external stressors that they're having to deal with, you know, relationships, job, injury, whatever the case may be, those are more the times that all implement an actual deload” (13).

“So, for my advanced athletes, [the deload is] more reactive rather than proactive. And then for my novice and intermediates, it's more proactive rather than reactive” (8).

## Discussion

The aim of this study was to explore strength and physique coaches' experiences of deloading and to enhance understanding of the organisation and management of resistance training deload training. This study is the first to document the strategies used by strength and physique coaches providing important contextual information for the understanding of deloading from the perspective of the strength and physique sport coach. Whilst results from this study are specific to strength and physique sports, several findings could have important implications for other sports that involve resistance training. Additional findings highlight the underrepresentation of deloading in the published literature, including a lack of operational definition, rationale, and organisation of training variables.

### How did participants define deloading and how was it differentiated from tapering?

Deloading was defined by participants as a short-term training cycle in which training demand is intentionally and systematically reduced. This finding is congruent with definitions provided elsewhere (22). However, future research should work towards a consensus definition of deloading to improve understanding of a commonly-used but under-researched training tactic, bridging the gap between research and practice, and standardising key terminology (65).

Although some participants considered deloading to be conceptually similar to tapering (in that both involve a reduction in training demand facilitated through manipulation of training volume or intensity of effort), deloading was considered a more flexible aspect of training that could occur at any point during the overall training programme. Previous research demonstrates that tapering occurs specifically in the days/weeks prior to competition (2), whereas deloading is likely to occur at the end of each training mesocycle (18, 24, 29). Therefore, the deload can be distinguished from the taper based on positionality within the training programme. Additionally, participants articulated that the deload can be distinguished from the taper based on objective, with the deload focusing on mitigating fatigue and not to “peak” performance. Participants described the deload as a sudden, nonprogressive reduction in training, where training demand remained constant throughout the duration of the deloading period. Whilst this approach is similar to a step taper, it is dissimilar to other conventional approaches to tapering, such as linear and exponential tapering, where training demand is reduced slowly or rapidly throughout the tapering period (66). Therefore, whilst deloading can be achieved through a nonprogressive decrease in training demand, tapering can be reduced in both a systematic linear and non-linear fashion (67).

Importantly, the terms *deload* and *taper* were used interchangeably by some participants, perhaps reflecting the similarities in structure between the two. Interestingly, all participants that used the terms interchangeably were involved in strength sports, where tapering is a common practice prior to competition. It is also evident that both terms have also been used interchangeably within the literature. For example, Wilson et al. (38) stated that the taper does not only take place prior to competition, but can take place at any point in the programme where the presence of overtraining is detected or during maintenance phases of training. Whilst this could be considered simple semantics, the interchangeability of terminology between the taper and deload could be confusing for both the practitioner and sports scientist who wish to better understand these concepts. Moreover, a lack of research exploring the optimal organisation of training during deloading might be due to misconceptions between the taper and deload.

### What was the purpose and rationale of deloading provided by participants?

Participants provided a rationale for the inclusion of deloading that related to fatigue management, recovery, and progression. Deloading was not rationalised as a tool to enhance performance *per se*. Instead, it was considered a short-term break from prolonged or challenging training to enhance readiness for the next training cycle. Additionally, participants suggested that the deload might mitigate the risk of injury or illness caused by prolonged or excessive resistance training. Colloquially, participants considered deloading to act as a “reset”, functioning as a physiological and psychological break from training that enabled preparedness for the athlete to “push again” in the next training phase. The objective of the deload is not to enhance performance, but instead to reduce fatigue that might impact the ability to train at the prescribed intensity of effort (14, 22). Further, it mitigates the risk of training maladaptation and injury (19). Indeed, previous research elucidated that prolonged or excessive resistance training can result in an unexplained reduction in performance indicative of non-functional overreaching and an increased risk of aches and pains (16, 68). Therefore, the inclusion of a deload might provide preventative benefits that mitigate the risk of maladaptation following prolonged periods of challenging resistance training.

Deloading might enhance preparedness for successive training cycles by reducing fatigue and monotony (23) whilst facilitating recovery and physiological adaptation following periods of strenuous training (18, 22, 24). Sports scientists and practitioners have postulated that deloading is important for overall progression within the context of periodisation for strength and muscle hypertrophy, and therefore intermittent use of lighter training periods may be important for overall athletic development (14, 15, 18, 24). There are very few studies that investigate the effects of continuous training (training over several weeks without deloading) versus periodic training (training followed by a detraining and retraining period). Research by Ogasawara et al. (69, 70) reported no differences in strength and muscle cross-sectional area (CSA) between a continuous training group and a periodic group (utilising three-week cessation after six weeks of training) over 15 and 24-week periods, despite the periodic group completing 20%–25% fewer workouts and thus training with lower total volume. As such, participants might have experienced a “resensitisation” effect where short-term detraining followed by retraining re-establishes anabolic signalling sensitivity (71). Results from this study revealed that participants, in part, programmed deloading to “*desensitise or reset the training stimulus”.* Indeed, mechanistic animal research (72) and, more recently, human research (71) have led to speculation that short-term training cessation might “refresh” blunted anabolic signalling caused by continuous resistance training. However, it is unclear whether this resensitisation effect would enhance muscle hypertrophy. It is worth noting that in both studies by Ogasawara et al. (69, 70), the training protocol consisted of a single exercise and participants were untrained, therefore it is uncertain whether such results would transfer to high-performance athletes undertaking resistance training programmes with multiple exercises. To date, there are no studies that have assessed the effects of deloading on muscle hypertrophy or strength compared to continuous training or training cessation.

### How did participants adapt training variables during deloading?

Participants defined deloading as a reduction in training demand, achieved through the adjustment of several training variables. Overall, participants emphasised an individualised athlete-centred approach to deloading and therefore spoke broadly about the adjustment of training principles during the deload, rather than providing iterative instructions. Deloading involved management and adjustment to training volume, intensity of effort, exercise selection, duration of training sessions and, in some, cases training frequency. Participants implemented deloading through a reduction in training volume (achieved through a decrease in either repetitions per set, sets per exercise or exercises per training session) or relative training intensity. Training volume was typically reduced by 30 to >50%, demonstrating a broad and individualised approach. In strength sports, it was common for the reduction in training volume to occur in non-specific accessory exercises, whereas in physique sports, the reduction in training volume was more flexible, typically manifesting as a reduction in both repetitions and sets per exercise across each training session. It was common for participants to reduce training intensity by modifying set endpoints (increased RIR/decreased RPE) and a reduction in repetition maximum. However, the decrease in repetition maximum was generally reserved for situations where a reduction in training volume and intensity of effort were not sufficient to achieve the desired outcome.

The information presented by participants in this study is reflected in the current literature, where deloading is achieved through a multifaceted alteration in training demand, facilitated through a reduction in training volume (18, 22, 25, 29, 31, 73) or training intensity of effort (29, 30, 32), as well as an alteration in exercise selection or order (25, 30). Previous research indicates that short-term, very low volume training can lead to meaningful increases in both strength and hypertrophy without detraining. For example, a multi-experiment research project investigating the minimum effective training dose for 1-RM strength reported that even in highly trained populations, 3–6 sets of 1–5 repetition sets per exercise per week may be enough to meaningfully increase 1-RM strength over 6–12 weeks (54). Previous research also indicates that as few as 1–3 sets per muscle group or exercise per week performed close or to momentary failure may be enough to induce significant increases in muscle hypertrophy or strength in both trained and untrained populations(74–78). However, given the lower intensity of effort employed during deloading, it is currently unclear how these variables should be organised and manipulated for adequate recovery without inducing a detraining effect. Moreover, further research should aim to investigate how these training variables can be organised to achieve optimal training outcomes.

Participants typically prescribed deloading for a period of 5 to 7 days. This was considered sufficient enough in duration to achieve a reduction in fatigue and to assist in recovery without athletes experiencing a detraining effect [“*a deload in excess of (6 days) likely means we're going to be reversing some of the adaptation”]*. However, previous research has elucidated that a decrease in muscular strength during short-term (<7 days) deloading is unlikely. A meta-analysis by Bosquet et al. (79) indicated that maximal force declined at a similar rate with a cessation period of <7 and 7–14 days, whilst the decrease only became significant after the third week of cessation. Whilst there is a considerable amount of literature investigating the effects of detraining on measures of muscular strength, by contrast, the effect of detraining on muscle hypertrophy is under-researched (80), especially in the short-term (i.e., <4 weeks). Peripheral adaptations, such as changes in muscle CSA and tendon properties, appear to decay faster than muscle strength and neural activation (81). For example, McMahon et al. (82) reported that muscle size (normalised physiological cross-sectional area allometrically scaled to body mass) reduced significantly (−6 ± 8%) during a 2-week period of cessation following 8-weeks of resistance training. The reduction in muscle size is in line with previous studies utilising measurements of anatomical CSA in recreationally active participants (83, 84). Conversely, Hwang et al. (85) reported no significant reductions in lean mass (utilising dual-energy x-ray absorptiometry) or rectus femoris CSA measured with ultrasound following a 2-week cessation period after a 4-week period of resistance training in resistance-trained individuals. Similarly, Vann (22) reported that in previously trained participants, neither 1-week of training cessation nor deloading led to significant losses in skeletal muscle size, indicated by both ultrasound and muscle fibre CSA analysis, after high-volume training. The discrepancy between such findings might be, in part, due to the difference in the training status of the participants, the instruments used to measure changes in muscle size, and the heterogeneous resistance training prescription between studies. Regardless, it is plausible that a training cessation period similar to the reported duration of a deload (i.e., <1 week), does not lead to relevant losses in muscle size or strength.

## Practical applications

This study is the first to explore deloading practices in strength and physique sports and provides important contextual information relating to deloading from the perspective of the coach. Based on the results of this study, a series of deloading guidelines have been created, aimed at assisting practitioners in the development of practical deloading strategies (see Table 3). Our findings note that coaches typically approach deloading flexibly and in an individualised manner, therefore, whilst the information presented is an interpretation of our findings and facilitates the successful planning and organisation of training and performance, coaches are encouraged to take a pragmatic approach, adapting the training programme to the needs of both the sport and athlete. Although the results of this study are specific to deloading in strength and physique sports, findings have implications for other sports where muscular strength and hypertrophy are important physical attributes.

**Table 3:** General guidelines for the prescription of deloading in strength and physique sports.

Training parameter	Adjustment(s) during deload
Training frequency	Whilst some practitioners might consider a reduction in training days during the deload, training frequency will typically remain unchanged (relative to normal training frequency).
Training volume	A reduction in training volume by approximately 30%–50%, achieved through a decrease in either repetitions per set or by a reduction in the number of sets per training session (or in some cases, both). Volume can be reduced in all exercises per session or by reducing the number of accessory exercises.
Intensity of effort	A reduction in the intensity of effort can be achieved by increasing proximity to muscular failure e.g., by adding 1–3 RIR for each set performed, by removing repetitions per set whilst maintaining absolute load or by reducing the absolute load (e.g., by 10%) whilst keeping the number of repetitions constant. Additionally (or in combination with an increase in proximity to muscular failure, a decrease in relative loading (e.g., 6 repetitions at 80% of 1-RM rather than 3 repetitions at 90% of 1-RM) can be implemented to facilitate the necessary reduction in the intensity of effort.
Exercise selection	The deload provides an opportunity to vary exercise selection as appropriate. Typically, sport-specific muscle groups/movements will remain in the training programme or should be exchanged for similar exercise movements. This will provide the athlete with some novelty and reduce monotony but maintain a level of training specificity. It should be noted that excessive changes in exercise selection might result in unwanted muscle soreness, therefore caution should be taken when making large alterations in programming.
Duration	Whilst it is important to approach the duration of the deload on an individual basis, most deloads will last 5 to 7 days.
Periodicity	For pre-planned training programmes, deloading can be scheduled every 4 to 8 weeks depending on the training demands of the mesocycle. This approach might be advantageous where individualised training is not possible (e.g., within a team or group environment). For training programmes that adopt an individualised, autoregulated approach, deloading should be approached flexibly, and integrated into the training programme only once sufficient objective and subjective data have been collected to justify changes to existing programming. In this sense, deloading should be prescribed as required. Periodicity of deloading is, in part, related to the preceding block of training i.e., deloading will be likely required after a period of overreaching but less likely during prolonged periods of continuous training where the overall training demand is relatively constant.

## Data Availability

The original contributions presented in the study are included in the article/**Supplementary Materials**, further inquiries can be directed to the corresponding author/s.
