# Supplementary material for: “You can't shoot another bullet until you've reloaded the gun”: Coaches' perceptions, practices and experiences of deloading in strength and physique sports
Source: Front Sports Act Living. 2022 Dec 21;4:1073223. doi: 10.3389/fspor.2022.1073223 (PMC9811819; doi:10.3389/fspor.2022.1073223)
Supplement: Supplementary file 1 [file Datasheet1.docx]

**Appendix 1: Interview Guide**

**INTRODUCTION**

- ***Introduction:*** *Introduce the study, your organization, and yourself*
- ***Inform:*** *Inform interviewee of 1) confidentiality, 2) anonymity, 3) right not to answer a question if they do not wish to, and 4) right to stop the interview at any time without jeopardy*
- ***Consent:*** *Get consent (verbal or written) to participate, and for the possible recording*
- ***Introduction questions*** *- tell me about yourself (make sure all descriptive questions are answered e.g., type of sport, experience duration, country of residence, the highest level of athlete relevant quals/accreditations, sports involved in, duration of time as a coach)*

**MAIN QUESTIONS**

**Broad questions:**

- How would you define or describe a deload?
  - ***Follow up:*** *How does it differ from taper/intro/pivot? (Ask for additional definitions as required)*
- Could you speak to me about your approach to deloads?
  - ***Aid if necessary:*** *WHAT, WHY, HOW, WHEN?*

**In-depth questions:**

1. What are some reasons you might incorporate deloads? - Are deloads necessary for making progress?
2. How regularly do you implement deloading? Are your deloads pre-scheduled or reactive to athlete needs? What drives that decision-making process?
3. What signs do you look for to tell you or the athlete is ready for a deload? Do you purposefully manipulate training demand (i.e., functionally overreach) before deload?
4. What variables do you manipulate when deloading? - Do you alter these based on the athlete’s level of competition (e.g., amateur vs. professional), age, or gender?
   - ***Follow up:*** *Do you introduce new training techniques/modalities/equipment during a deload? How about nutritional changes?*
5. What does a successful deload look like? How about non-successful? - When and how does an athlete resume training after deload?

**Clarifications and link to theory:**

- Do your athletes enjoy deloads? Why? / Why not?
- What are your primary sources of information for deloads? What would you like to know about deloads in the future?

**CLOSING**

- Closing question/statement - is there anything you’d like to add or go over again? (Provide an opportunity to recap or re-answer any questions)
- Collect demographic information
- Thank the respondent
- Inform them of what will happen after the interview
- Provide contact information if they need to contact the organisation about the study
